# 875-A. Psychiatric Sequelae of Burn Injury: Comparing Male and Female Survivors

**DOI:** 10.1093/jbcr/irag033.353

**Published:** 2026-04-06

**Authors:** Blancheneige M Beohon, Laeticia P Evang, Pekam Njowo, Chikobi Ezenwukwa, Kelly Georgestone

**Affiliations:** University of Texas Medical Branch, Galveston, TX; University of Texas Medical Branch, Galveston, TX; University of Texas Medical Branch John Sealy School of Medicine, Galveston, TX; University of Texas Medical Branch, Galveston, TX; University of Texas Medical Branch, Galveston, TX

## Abstract

**Introduction:**

Burn injuries are associated with significant long-term physical and psychological morbidity. Emerging evidence suggests that psychiatric sequelae following burns may differ by sex, yet little is known about these differences among Black burn survivors, a population disproportionately affected by health inequities. This study aimed to evaluate sex-based differences in post-burn psychiatric comorbidities, including post-traumatic stress disorder (PTSD), anxiety, depression, suicidality, and substance use disorder.

**Methods:**

A retrospective cohort analysis was conducted using the TriNetX Research Network, a national federated electronic health record database. Black male and female patients with burn injury were identified using ICD-10 codes. Cohorts were stratified by sex, and outcomes assessed included new diagnoses of PTSD, anxiety disorders, depressive disorders, suicidal ideation/attempt, and substance use disorders and were assessed at 30 days. Patients with pre-existing diagnoses were excluded. Risk ratios (RR) were calculated with 95% confidence intervals (CI). Statistical significance was deemed *p*<.05.

**Results:**

Black females were at significantly higher risk of anxiety (RR 1.50, *p*<.0001) and depression (RR 1.29, *p*=.0019) compared to Black males. In contrast, Black males were at higher risk of suicide (RR 0.67, *p*=.0099) and substance use disorder (RR 0.49, *p*<.0001). Although not significant, a lower risk in differences were observed for PTSD (RR 0.79, *p*=.0861).

**Conclusions:**

Black female burn survivors demonstrated significantly higher risk of anxiety and depression, while Black male survivors exhibited higher risk of suicidality and substance use disorder. Rates of PTSD were similar between sexes. Future studies should expand this analysis to other racial groups to determine whether these disparities are driven primarily by sex or by intersecting effects of sex and race.

**Applicability of Research to Practice:**

These findings highlight the importance of tailoring post-burn psychiatric screening and interventions by sex within the Black burn survivor population. Early, sex-specific psychosocial interventions may improve long-term outcomes and reduce disparities in care.

**Funding for the study:**

Supported by UTMB Institute for Translational Sciences (UL1 TR001439), funded by the National Center for Advancing Translational Sciences at the NIH.

